# Influence of coil orientation on corticospinal excitability of trunk muscles during postural and volitional tasks in healthy adults

**DOI:** 10.3389/fnhum.2023.1108169

**Published:** 2023-02-01

**Authors:** Wesley Ma, Sheanil Nemdharry, Edith Elgueta Cancino, Shin-Yi Chiou

**Affiliations:** ^1^School of Sport, Exercise and Rehabilitation Science, University of Birmingham, Birmingham, United Kingdom; ^2^Exercise and Rehabilitation Sciences Institute, School of Physical Therapy, Faculty of Rehabilitation Science, Universidad Andrés Bello, Santiago, Chile

**Keywords:** transcranial magnetic stimulation, motor evoked potentials, erector spinae, anticipatory postural adjustments, static contractions, electromyography

## Abstract

**Introduction:**

Trunk muscles play a role in maintaining postural stability and performing goal-directed voluntary movements in activities of daily living. Evidence has shown that the primary motor cortex (M1) is involved in modulation of postural control and voluntary movements of the trunk. However, it remains unknown whether the neural circuits within the M1 were recruited to the same extent between a postural task and a goal-directed voluntary task.

**Methods:**

To address this, we examined latencies and amplitudes of motor evoked potentials (MEPs) of the erector spinae (ES) with transcranial magnetic stimulation (TMS) figure-of-eight coil oriented to induce latero-medial (LM), posterior-anterior (PA), and anterior-posterior (AP) currents in the M1 in twenty healthy participants during a dynamic shoulder flexion (DSF) task, a postural task requiring anticipatory postural adjustments (APAs), and during a static trunk extension (STE) task, a voluntary task without involvement of APAs.

**Results:**

We found that differences in the AP-LM latency of ES MEP were longer compared with the PA-LM latency in both tasks. Corticospinal excitability was overall greater during the DSF task than during the STE task irrespective of the coil orientation.

**Discussion:**

Our findings suggest that while the same neural circuits in the M1 were recruited to modulate both postural and voluntary control of the trunk, the contribution was greater to the postural task than the voluntary task, possibly due to the requirement of APAs in the task.

## 1. Introduction

Trunk muscles are activated during goal-directed movements, such as flexion and extension (Thorstensson et al., 1985; Oddsson and Thorstensson, 1986), as well as during movements of the upper extremities, i.e., a fast bilateral shoulder flexion movement, for maintaining postural stability (Aruin and Latash, 1996; Hodges and Richardson, 1997). Research has shown that the primary motor cortex (M1) and the corticospinal tract are involved in neural control of the trunk muscles (Masse-Alarie et al., 2012, 2018; Chiou et al., 2016, 2018). Previous studies reported increased amplitudes of motor evoked potentials (MEPs) elicited by transcranial magnetic stimulation (TMS) placed over the M1 during voluntary contractions of the erector spinae (ES) muscles (Ferbert et al., 1992; Nowicky et al., 2001; Chiou et al., 2016), in keeping with the notion that the corticospinal tract contributes to voluntary movement (Lemon, 2008). For postural control, the two mechanisms commonly observed in the trunk muscles are anticipatory postural adjustments (APAs) and compensatory postural adjustments (Kanekar and Aruin, 2014a). APAs are often initiated within a timeframe from 100 ms prior to the onset of the prime mover to 50 ms after the onset (Aruin and Latash, 1996). Because the timeframe is considered to be too early for the afferent input from the periphery to reach to the M1 (Friedli et al., 1984; Massion, 1992), they are thought to be pre-planned and mediated by the M1. Indeed, prior work has revealed increased motor cortical excitability of the ES muscles prior to the onset of the bilateral shoulder flexion movement, i.e., during the APA window, in healthy adults (Masse-Alarie et al., 2012; Chiou et al., 2016; Rowland et al., 2021). Furthermore, studies comparing postural and voluntary tasks reported greater cortical contribution to the trunk muscles when muscle activity was matched across the tasks (Guz, 1997; Chiou et al., 2016), suggesting distinct motor cortical circuits mediating postural and volitional tasks of the trunk.

Several lines of evidence have shown that stimulating the M1 with TMS can activate corticospinal neurons which elicit multiple volleys through the corticospinal tract that results in a MEP in a target muscle in humans (Day et al., 1989; Di Lazzaro et al., 1998, 1999). Depending on the current flow across the motor representation of the M1, TMS is likely to evoke different set of synaptic inputs to corticospinal neurons (Di Lazzaro et al., 2012; Di Lazzaro and Rothwell, 2014). Evidence in humans with epidural recordings of descending activity in the corticospinal tract revealed that the posterior-anterior (PA) currents preferentially generate early indirect-waves (I1-waves), whilst the anterior-posterior (AP) currents preferentially generate late I-waves (I2-I3-waves) that occur 1.2–1.5 ms after the I1-waves (Di Lazzaro et al., 1998, 1999; Di Lazzaro and Rothwell, 2014). It is thought that the early and late I-waves represent two sets of motor cortical circuits that have distinct contributions to human motor control (Hamada et al., 2014; Federico and Perez, 2017; Hannah et al., 2018). For instance, when applying different currents at different phases of a voluntary movement, research found that the excitability of the late I-waves, not the early I-waves, correlated with the scale of voluntary contractions (Kurz and Leukel, 2019), whereas neither early nor late I-waves seem to be affected at the onset of the voluntary movement (Hannah et al., 2018; Kurz and Leukel, 2019).

The influence of TMS-induced current directions on corticospinal excitability of the trunk muscles is less understood. Using single and paired-pulse TMS paradigms a previous study reported that the AP currents generated longer MEP latencies and greater motor cortical inhibition of the ES muscles during the voluntary trunk extension, compared with the PA currents (Desmons et al., 2021). However, it remains unclear the extent to which motor cortical circuits that are preferentially activated by different current direction contributed postural and voluntary control of the ES muscles. Hence, the aim of the study was to examine influences of separate motor cortical circuits, activated by PA and AP currents, on corticospinal excitability of the ES muscles during a postural task requiring APAs and during a volitional task without APAs in healthy adults. Given different corticospinal excitability of the ES muscle between a postural task and a volitional task of the trunk (Chiou et al., 2016), we hypothesized that differences between MEPs elicited by PA and AP currents in the ES muscle during the postural task requiring APAs would be different from that during the volitional task with minimum APA involvement in healthy adults. To test our hypothesis, we examined the MEP latencies and amplitudes elicited by PA and AP currents in the ES muscle during a rapid shoulder flexion task and a static trunk extension task and compared them with those elicited by a lateral-medial (LM) current at a higher intensity which is thought to directly activate the axons of the corticospinal tract, thus by-passing the M1 (Patton and Amassian, 1954).

## 2. Materials and methods

### 2.1. Participants

The study received ethical approval from the School of Sport, Exercise and Rehabilitation Sciences Ethics Committee at the University of Birmingham (MCR2122_15) in accordance with the guidelines established in the Declaration of Helsinki. Twenty healthy participants (13 males: 7 females, 19 right-handed, mean age: 24 ± 3 years) were recruited from staff and students at the authors' institution. Exclusion criteria consisted of a contraindication to TMS (e.g., a history of epilepsy, syncope, contain metal devices, or implants in the brain) (Rossi et al., 2011), currently pregnant, musculoskeletal injuries to the upper limbs or trunk, or no visible MEP elicited by TMS in the ES muscle. All participants provided written informed consent prior to any data collection.

### 2.2. Electromyography

Surface electromyography (EMG) was measured bilaterally from the anterior deltoid (AD) and ES muscles at the 12th thoracic vertebral level (T12). The skin was cleaned and prepared prior to application of pairs of Ag/AgCl electrodes (self-adhesive, 2 cm diameter, CareFusion, UK). The electrode pairs were placed on the muscle belly of the specified muscles parallel to the direction of muscle fibers with a 2 cm inter-electrode distance. The ground electrode was placed over the spinous process at the level of C7. EMG signals were amplified (Digitimer D360, 1,000×), filtered (10–1,000 Hz) and sampled at 2,000 Hz with a Micro1401-4 data acquisition system (Cambridge Electrical Design, UK). Raw EMG data was recorded with Signal (Version 6.06) software and stored on a password protected computer.

### 2.3. Transcranial magnetic stimulation

**Figure 1:**
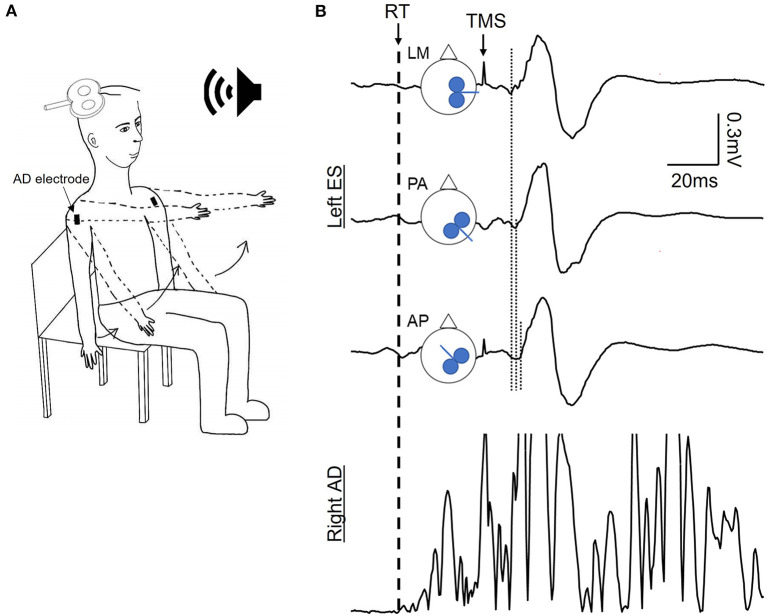
Experimental setup. **(A)** Schematic of the dynamic shoulder flexion (DSF) task initiated in response to a verbal cue. **(B)** Schematic of coil orientation of the transcranial magnetic stimulation (TMS) and examples of motor evoked potentials (MEPs) averaged from 15 MEPs in the contralateral erector spinae (ES) muscle and rectified EMG traces in the anterior deltoid (AD) during the DSF task. Dash line indicating the reaction time of the DSF task according to the EMG onset of AD; dotted lines indicating the onset latency of ES MEP elicited by the TMS coil oriented to induce the latero-medial (LM), posterior-anterior (PA), and anterior-posterior (PA) currents in the brain. Note that the MEP latency elicited by the AP and PA currents were longer compared with the LM current.

Monophasic pulses were delivered from The Magstim 200^2^ stimulator (Magstim, Whitland, UK) through a figure-of-eight coil (model Magstim D70^2^). The hotspot for stimulation was determined as the location on the scalp where the largest peak-to-peak amplitude of MEP response of the contralateral ES muscle was found. For consistency, right hemisphere was targeted in identifying the hotspot for the left ES muscle in all participants. While searching for the hotspot, the coil was positioned at 45° away from the midline with the handle pointing posteriorly. Once the hotspot was located the active motor threshold (AMT) was identified as the lowest intensity required to elicit a minimum of three visible MEPs within six consecutive stimulations (Chiou et al., 2018; Rowland et al., 2021) while participants were sitting upright in chair. Amplitudes of the background EMG was monitored by an experimenter and verbal feedback was provided to the participant to ensure a consistent activation of the ES muscle during the determination of the AMT. AMTs were individually determined for each of the three coil orientations: latero-medial (LM), posterior-anterior (PA), and anterior-posterior (AP) directions (Figure 1B). The LM orientation the coil was rotated 45° medially compared to the PA orientation, directing the current flow directly toward the midline of the head; the AP orientation positioned the coil 180° from the PA orientation with the handle pointing anteriorly. The position of the coil at each direction was recorded with a navigation system (Brainsight, version 2.4.8, Rogue Research Inc., Canada) to ensure accuracy of the coil position throughout each direction and the experiment. The intensity of TMS at each orientation was calculated to be 1.2 × AMT for the PA and AP orientations, and 1.5 × AMT in LM orientation (Hamada et al., 2013; Federico and Perez, 2017). A higher stimulus intensity used for LM was to ensure that corticospinal neurons were directly activated at this coil orientation (D-wave; Di Lazzaro and Rothwell, 2014). Fifteen stimuli were delivered at each coil orientation.

### 2.4. Experimental procedures

Participants were instructed to perform two tasks: bilateral dynamic shoulder flexion (DSF) and static trunk extension (STE) in an upright seated posture. In the DSF task, participants were instructed to flex both shoulder joints from 0 to 90° as fast as possible in response to a verbal “Go” cue from the experimenter without flexing the elbows or wrists (Figure 1A). The interval of the verbal cues was varied to avoid anticipation of the participants to the task. A threshold-crossing feature of Signal was applied to the rectified EMG traces of the AD ipsilateral to the TMS coil during the DSF task to detect the visible rise in the EMG amplitude of the AD (i.e., EMG onset of AD) which subsequently triggered the delivery of the TMS pulse with a 25-ms delay (Chiou et al., 2016). This timing was to ensure that the TMS stimuli were delivered during the APA window of the ES muscle (Aruin and Latash, 1996; Tsao et al., 2008). Fifteen successful trials were recorded per each coil orientation from each participant. For the STE task, participants were instructed to sit upright and voluntarily contract the ES muscles to a level that matched to the EMG activity during the DSF task. To do so, the EMG of the ES muscle contralateral to the TMS from the DSF task were firstly rectified and the mean amplitude was calculated in a 25-ms window prior to the stimulus. The level of EMG activity was then displayed on a screen placed in front of the participants. Participants were instructed to perform a static, sustained contraction (~1.5 s) of the ES muscle to the same level of the EMG activity prior to a TMS pulse and to relax after the TMS. Participants repeated the task until 15 trials with the matched pre-stimulus EMG activity were obtained. For the purposes of EMG normalization, participants performed three brief (~2 s) maximal voluntary contractions (MVCs) of the trunk extensors in a prone position on a plinth with the pelvis and the legs strapped securely and resistance provided at the scapulae. Consistent verbal encouragement was given during the MVCs.

### 2.5. Data analysis

All EMG recordings were visually inspected and frames with unsuccessful performance (e.g., anticipating response, unmatched ES activity) or no clear MEP were removed. Fifteen MEPs were averaged for each coil orientation in each task. Peak-to-peak amplitudes of the averaged MEPs were measured to present corticospinal excitability of the ES muscle. EMG traces obtained during the tasks were rectified and pre-stimulus background EMG was calculated as mean amplitudes in 25 ms window and 150 ms window prior to the stimulus artifact in the DSF and STE tasks, respectively. The background EMG of the ES muscle was normalized to the MVC and expressed as a percentage of the mean amplitude of MVC. For MEP latencies, individual frames in each coil orientation and in each task were measured manually for each participant by the same researchers (WM and SN) for consistency. The onset latency of MEP was determined as the point where rectified EMG traces exceeded 2 SD of the mean pre-stimulus EMG level (Hodges and Richardson, 1997; Chiou et al., 2016). When a latency was unable to be clearly defined from the rectified trace due to ongoing EMG activity in the ES muscle, we compared the rectified and unrectified traces and determined the MEP latency as accurate as possible. Differences in MEP latencies between PA and LM coil orientation as well as between AP and LM coil orientation were also calculated.

### 2.6. Statistical analysis

The collected data were analyzed using Statistical Program for the Social Sciences (SPSS, version 28.0, IBM Corp). Normal distribution was tested by the Shapiro-Wilk test; all variables passed the normality tests (*p* > 0.05) and hence parametric tests were applied. The Mauchly test was used to test sphericity; when the sphericity assumption failed, the Greenhouse-Geisser correction statistic was applied. Two-way repeated-measures ANOVAs were performed to examine the effect of coil orientation (LM, PA, and AP) and task (DSF and STE) on MEP latencies, differences in MEP latencies, MEP amplitudes, and background EMG. A repeated-measures ANOVA was applied to determine the effect of coil orientation on the AMT. When there was a main effect, a *post-hoc* analysis was applied. Statistical significance of tests was *p* < 0.05 and *p*-values were corrected using the Bonferroni correction for multiple comparisons if needed. Results were presented as mean ± SD in the text.

## 3. Results

### 3.1. Active motor threshold of the ES muscle in different coil orientations

Repeated measures ANOVA showed a main effect of coil orientation on the AMTs of the ES muscle (*F*_2,38_ = 26.69, *p* < 0.001). *Post-hoc* analysis revealed that the AMT was higher in the AP direction (71.5 ± 7.37%) than in the PA (62.3 ± 6.93%; *p* < 0.001) and LM (63.65 ± 7.82%; *p* < 0.001) directions; the AMTs were the same when the coil was held in the PA and LM directions (*p* = 0.25).

### 3.2. MEP latencies

**Figure 2:**
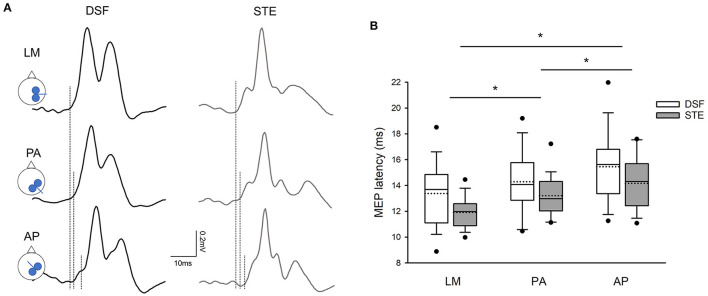
MEP latencies. **(A)** Averaged MEPs from 15 rectified EMG traces in the contralateral erector spinae (ES) elicited by different coil orientations during the dynamic shoulder flexion (DSF) task and during the static trunk extension (STE) task in a representative participant. Note that the latency of ES MEPs elicited by the AP current direction was the longest, followed by the PA and LM current directions in both DSF and STE tasks. **(B)** Group mean data (*n* = 20) demonstrating the onset latencies of ES MEPs with all current direction. Solid lines indicate median values; dotted lines indicate mean values. The box is interquartile range; error bars denote maximum and minimum values. *p < 0.05 between coil orientation.

Figure 2A illustrates the averaged MEP latencies in a representative participant during the DSF and STE tasks with the coil in the LM, PA, and AP directions. Note that the MEP latency elicited with the coil was the shortest in the LM direction, followed by PA direction and AP direction in both DSF and STE tasks. This was confirmed by the group results demonstrating a main effect of coil orientation (*F*_2,38_ = 29.72, *p* < 0.001) and task (*F*_1,19_ = 8.02, *p* = 0.011), but not in their interaction (*F*_2,38_ = 0.458, *p* = 0.64). *Post-hoc* tests showed that overall MEP latencies elicited with the coil in the LM direction (12.64 ± 2.05 ms) were shorter compared to the PA (13.75 ± 2.05 ms; *p* < 0.001) and AP (14.81 ± 2.51 ms; *p* < 0.001) directions, and the MEP latency was shorter in the PA direction than the AP direction (*p* < 0.001; Figure 2B). Furthermore, the overall MEP latencies were shorter in the STE task (13.09 ± 1.83 ms) than in the DSF task (14.37 ± 2.67 ms; *p* = 0.011).

**Figure 3:**
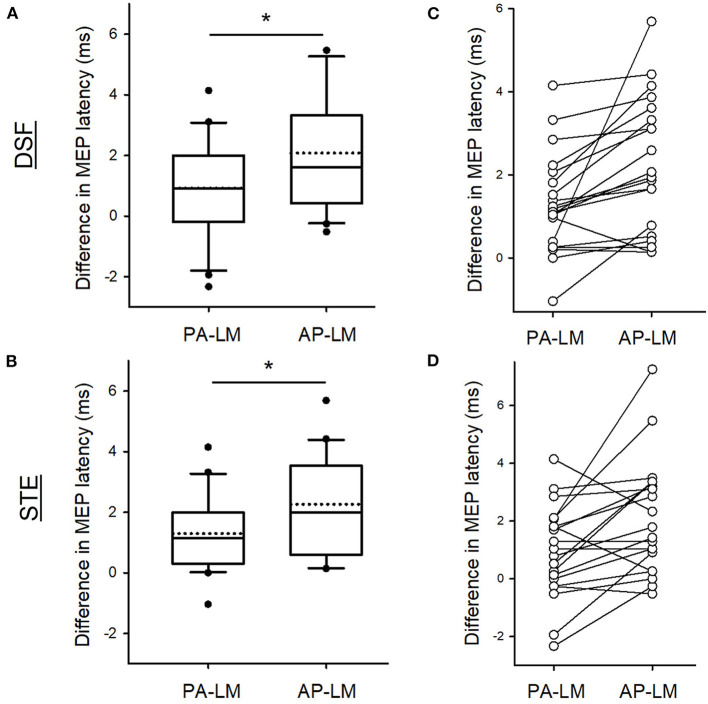
Differences in MEP latencies between coil orientations. Group data showing the differences in the MEP latencies between the PA and LM and between AP and LM coil orientation during the DSF **(A)** and during the STE **(B)**. Individual data demonstrating that the majority of the participants having a longer AP-LM latency compared with PA-LM latency in both tasks **(C, D)**. Solid lines indicate median values; dotted lines indicate mean values. The box is interquartile range; error bars denote maximum and minimum values. *p < 0.05 between conditions.

When comparing MEP latencies elicited by the PA and AP coil orientation with the LM coil orientation, there was a main effect of coil orientation (*F*_2,38_ = 13.754, *p* < 0.001), but not task (*F*_2,38_ = 0.42, *p* = 0.52) or their interaction (*F*_2,38_ = 0.34, *p* = 0.57). Group results revealed that the differences in the latencies of MEP were greater between AP-LM coil orientation (2.17 ± 1.79 ms) than between PA-LM coil orientation (1.11 ± 1.42 ms; *p* = 0.001; Figures 3A, B). Note that the majority of the participants showed a longer MEP latency elicited by the AP direction with respect to the LM direction in comparison to that by the PA direction in relation to the LM direction in both tasks (Figures 3C, D).

### 3.3. Motor evoked potentials

**Figure 4:**
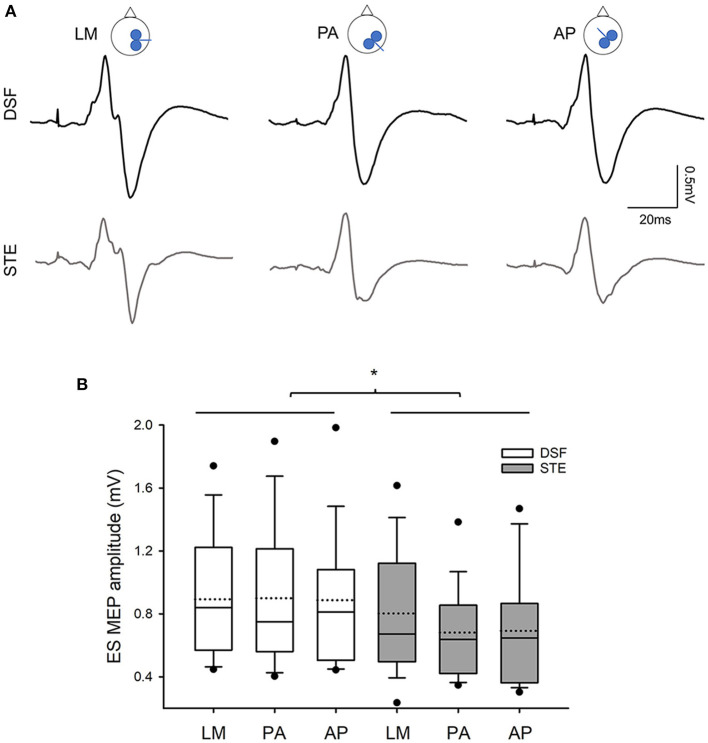
MEP amplitudes **(A)** Averaged MEPs from 15 raw EMG traces in the contralateral ES muscle elicited by different coil orientations during the DSF and STE tasks in a representative participants. Note that MEP amplitudes are greater during the DSF task than during the STE task in all three coil orientations. **(B)** Group data (*n* = 20) showing amplitudes of ES MEPs in three coil orientations during the DSF and STE tasks. Solid lines indicate median values; dotted lines indicate mean values. The box is interquartile range; error bars denote maximum and minimum values. *p < 0.05 between conditions.

Figure 4A illustrates the average of 15 EMG traces of MEPs in the ES muscle in a representative participant during the DSF and STE tasks with the different coil directions. Note that the MEP size is greater during the DSF task than during the STE task. Repeated measures ANOVA revealed a main effect of task (*F*_1,18_ = 4.62, *p* = 0.045) but no effect of coil orientation (*F*_2,36_ = 1.15, *p* = 0.33) or their interaction (*F*_2,36_ = 1.88, *p* = 0.17) on the MEP size. Overall, the amplitudes of ES MEPs were greater during the DSF task (0.89 ± 0.41 mV) than during the STE task (0.72 ± 0.34 mV; Figure 4B). Furthermore, results revealed no effect of Coil (*F*_2,23_ = 1.203, *p* = 0.312), Task (*F*_1,19_ = 0.338, *p* = 0.568) or their interaction (*F*_2,38_ = 2.006, *p* = 0.148) on the background EMG amplitudes in the ES muscle (DSF: 35.96 ± 12.52% MVC; STE: 34.78 ± 16.80% MVC), suggesting that activity of the ES muscles was the same across all conditions.

## 4. Discussion

Our results indicate that the ES muscle receive innervations from two separate corticospinal neurons that are preferentially activated by PA and AP currents during the DSF task and the STE task and the extent to which the two motor neuronal circuits contributed to modulation of the postural and voluntary tasks was the same. We observed a longer onset latency of ES MEP elicited by the TMS coil orientated to induce the AP current in the M1 than the PA current with respect to the LM current in both DSF and STE tasks, suggesting that the early and late I-waves engaged to the same extent during both tasks. Furthermore, corticospinal excitability of the ES muscle was greater during the DSF task than during the STE task irrespective of the coil orientation, contrary to our hypothesis. Based on these findings, we proposed that the two sets of motor cortical circuits elicited by PA and AP currents both contribute to APAs over volitional control of the trunk.

### 4.1. PA and AP-induced currents in the brain in APAs and goal-directed movement of the trunk

There is little understanding of TMS-induced currents in the M1 recruiting different corticospinal neurons projecting to the trunk muscles in humans. Our knowledge in PA- and AP-related synaptic inputs in human motor control is largely from research in muscles of the upper extremities during voluntary movements (Hamada et al., 2014; Federico and Perez, 2017; Hannah et al., 2018; Kurz and Leukel, 2019). Hence, the first question to address in our study was to determine whether characteristics of ES MEP were different between PA and AP-induced currents as shown in the previous studies. We found that the AMT was higher when the coil was oriented to induce the AP current than that to induce the PA current in the M1. This agrees with prior work investigating the TMS-induced currents in hand muscles with a figure-of-eight coil (Sakai et al., 1997; Cirillo and Byblow, 2016; Sale et al., 2016) as well as with a previous study using a double-cone coil in the lumbar ES muscles (Desmons et al., 2021) reporting lower motor thresholds with the PA current than the AP current. Additionally, our results obtained in the STE task revealed that the latency of ES MEP was 1.3 and 2.3 ms longer in the PA current and in the AP current compared with the latency elicited by the LM current during a sustained voluntary contraction of the ES muscle, in keeping with the differences in MEP latencies of early and late I-waves with respect to the D-wave from the epidural recordings (Di Lazzaro et al., 1998, 2012) and from surface EMG recordings (Sakai et al., 1997; Hamada et al., 2013; Volz et al., 2015). The MEP latencies during the DSF task were the same as during the STE task, albeit they were slightly shorter; the differences were 0.92 and 2.08 ms in the PA and AP currents with respect to the LM current during the DSF task, respectively. Prior work reported variable differences of 2.7–3.2 ms (Hamada et al., 2013; Wiethoff et al., 2014; McCambridge et al., 2015; Volz et al., 2015), with a range from 0.5 to 6 ms, in the MEP latency between the AP and LM currents. Our results from the DSF and STE tasks were within this range and in agreement with the evidence that MEPs elicited by the TMS-induced PA current preferentially activate the early I-waves, whilst the response elicited by the AP current preferentially activate the late I-waves (Di Lazzaro et al., 2012; Di Lazzaro and Rothwell, 2014). Taken together, our findings revealed that there are two separate motor cortical circuits projecting to the ES muscle that can be probed using the TMS-induced PA and AP currents in humans, in keeping with the research evidence reported in the hand muscles. Furthermore, despite different methodologies employed, our results support a previous study probing the corticospinal projecting to the lumbar ES muscle with the double-cone coil (Desmons et al., 2021). The motor threshold is often higher in the trunk muscles than in the hand muscles and as such a double-cone coil is more likely to evoke visible and consistent MEPs in these muscles. Our study suggests that both types of TMS coils are suitable for investigations of different synaptic inputs related to early and late I-waves in trunk motor control.

We found that overall corticospinal excitability was greater during the DSF task than during the STE task, in keeping with our previous study comparing MEP amplitudes elicited with the PA current during different postural tasks (Chiou et al., 2016). An intriguing question is why the current direction had minimal influences on corticospinal excitability between the DSF and STE tasks, as opposed to our hypothesis? Previous investigations using directional-TMS method in healthy adults showed that the excitability of the early and late I-waves, activated by the PA and AP currents of TMS, respectively, was unaffected at the onset of a voluntary movement (Hannah et al., 2018; Kurz and Leukel, 2019), possibly due to lack of afferent input from the periphery at the movement onset integrating with the motor commands in the M1. During the DSF task, the stimulation occurring at 25 ms after the onset of shoulder flexion within the APA window was considered to be too early for any sensory input (e.g., proprioception) to reach to the M1 (Fetz et al., 1980). As a result, the amplitudes of ES MEPs induced by the PA and AP currents were the same, in line with the previous findings showing similar excitability of early and late I-waves at the movement onset (Hannah et al., 2018; Kurz and Leukel, 2019). In contrast to the DSF task, the STE task comprised a sustained voluntary contraction of the ES and the stimulation was delivered during the contractions, thereby sufficient time for the arrival of the afferent input to the M1. Prior work in humans reported a correlation between the excitability of the late I-waves and the levels of force during isotonic muscle contractions of a small hand muscle to different force levels; the excitability of the early I-waves was however unaffected by the force (Kurz and Leukel, 2019). This is different from our results which showed no difference in MEP size between PA and AP currents during the STE task. However, research recording corticospinal neurons with invasive procedures in monkeys during precision and power grips reported that activity of the corticospinal neurons was unaffected by difference forces during the power grip but only modulated by the precision grip (Muir and Lemon, 1983), indicating that these cells were associated with motor control but not with changes in force. Moreover, work using a similar methodology as to our study also reported no change in MEP recruitment curve when it was elicited by the PA or the AP current with a double-cone coil during sustained voluntary contractions of the lumbar ES muscles (Desmons et al., 2021). The different results may reflect different neural control between the hand and trunk muscles and further research is required to determine the role of late I-waves in motor control of the trunk.

### 4.2. Functional considerations

Altered trunk control is common in older adults (Kanekar and Aruin, 2014b) and in people with neurological conditions, such as spinal cord injury (Milosevic et al., 2015), Parkinson's Disease (Latash et al., 1995), and stroke (Dickstein et al., 2004). Evidence has suggested an association between altered trunk control and changes in corticospinal function; for example, reduced facilitation in the ES muscle during voluntary contractions of elbow flexors correlated with delays in APAs in individuals with spinal cord injury (Chiou and Strutton, 2020), and corticospinal excitability prior to the onset of shoulder flexion was associated with delays in the onset of EMG activity of the ES muscle in older adults (Rowland et al., 2021). Our study demonstrates the possibility of evaluation the corticospinal function using non-invasive, directional-TMS method in the populations with impaired trunk control that may aid to differential diagnosis and selection of tailoring rehabilitation and neuromodulatory techniques for treating impairment of the trunk muscles. For instance, by pairing electrical muscle stimulation with auditory stimuli during normal daily activities for ~6 h, work has shown improved reaction time and enhanced motor responses elicited by a TMS-induced AP current, but not by the PA current in healthy adults (Germann and Baker, 2021), suggesting targeted neuroplasticity induced by the paired stimulation. There are a range of interventions available for trunk rehabilitation from conventional activity-based exercise (Eginyan et al., 2021) and electrical muscle stimulation (Bheemreddy et al., 2020) to innovative transcutaneous spinal cord stimulation (Roberts et al., 2021). Further research is required to determine the effect of therapeutic modalities on corticospinal neurons generating early and late I-waves projecting to the trunk muscles to better inform targeted treatment for individuals.

### 4.3. Limitations

There are limitations in this study. Firstly, we chose an intensity of 1.5× AMT for the TMS coil orientation to induce the LM current in the brain based on the literature that a high intensity is likely to directly activate corticospinal axons (D-waves) which generates a response with the shortest response latency (Di Lazzaro and Rothwell, 2014). Due to a combination of a higher motor threshold of the trunk muscle and the choice of the coil, 6 out of 20 participants in our study had a testing intensity for the LM coil direction to be more than 100% MSO and hence were stimulated with an intensity of 100%MSO instead. This may affect the results of the onset latency, particularly when comparing the MEP latencies of the PA and AP currents with the latency of the LM current. Given our results were consistent with the previous findings using a double-cone coil in the lumbar ES muscles, this limitation may be overcome by a different choice of the TMS coil. Another limitation is that we used an intensity at 1.2× AMT which was higher than the intensity used in previous studies investigating the I-waves in the hand muscles (Hamada et al., 2013; Federico and Perez, 2017). Due to ongoing background EMG in the ES muscle during the tasks, a lower intensity was less likely to evoke visible MEPs clear from the background EMG activity. It is suggested that an increase in stimulus intensity in the PA coil direction could potentially activate the late I-waves, in additional to the early I-waves (Di Lazzaro et al., 2012; Di Lazzaro and Rothwell, 2014). However, the difference in the MEP latency elicited with the PA coil direction with respect to the LM coil direction was 1.1 ms within the range reported in the literature (Di Lazzaro and Rothwell, 2014; Opie and Semmler, 2021), although we were unable to completely rule out the possibility of some late I-waves being activated with the PA current. Finally, it is possible that the greater corticospinal excitability of the ES during the DSF task compared with the STE task reflected neural control of dynamic muscle contractions (Arányi et al., 1998). However, activation of the ES muscle during the DSF task was to maintain the upright posture and our results were in line with a previous study which controlled for the phasic movement of the trunk during the DSF task. Nevertheless, since we did not restrain the trunk but instructed the participants to keep their torso still during the DSF task, the contribution of the neural mechanisms of the dynamic task to the corticospinal excitability cannot be fully excluded.

## 5. Conclusions

Our findings demonstrate the latencies of ES MEP elicited by a TMS coil oriented to induce the AP current were longer compared with the PA current, suggesting that the ES muscle receives different synaptic inputs through the corticospinal tract. The synaptic inputs activated by the PA and AP currents contributed to the same extent in both postural and goal-directed voluntary tasks. Additionally, corticospinal excitability was greater during the postural task requiring APAs than during the voluntary task regardless the TMS coil oriented differently, suggesting greater involvement of the two sets of motor cortical circuits in a task requiring APAs. Our study provides new knowledge in motor cortical involvement in trunk motor control in humans.

## Data availability statement

The datasets presented in this article are not readily available because participant's data needs to be handled in accordance with the current data protection laws and ethical guidelines. Requests to access the datasets should be directed to s.chiou@bham.ac.uk.

## Ethics statement

The studies involving human participants were reviewed and approved by University of Birmingham School of Sport, Exercise, and Rehabilitation Sciences Research Ethics Committee, MCR2122_15. The patients/participants provided their written informed consent to participate in this study.

## Author contributions

S-YC and EE: study concept and design and editing the manuscript and figures. WM, SN, EE, and S-YC: data acquisition and analysis. WM, SN, and S-YC: drafting the manuscript and figures. All authors read and approved the final version of this manuscript.
